# Virulence of *Trypanosoma cruzi* Strains Is Related to the Differential Expression of Innate Immune Receptors in the Heart

**DOI:** 10.3389/fcimb.2021.696719

**Published:** 2021-07-15

**Authors:** Tamyres Bernadete Dantas Queiroga, Nathalie de Sena Pereira, Denis Dantas da Silva, Cléber de Mesquita Andrade, Raimundo Fernandes de Araújo Júnior, Carlos Ramon do Nascimento Brito, Lúcia Maria da Cunha Galvão, Antônia Cláudia Jácome da Câmara, Manuela Sales Lima Nascimento, Paulo Marcos Matta Guedes

**Affiliations:** ^1^ Graduate Program Parasitary Biology, Federal University of Rio Grande do Norte, Natal, Brazil; ^2^ Graduate Program Health and Biological Sciences, Federal University of Vale do São Francisco, Petrolina, Brazil; ^3^ Department of Biomedical Sciences, Rio Grande do Norte State University, Natal, Brazil; ^4^ Laboratory of Investigation of the Inflammation and Cancer (LAICI)/Department of Morphology, Federal University of Rio Grande do Norte, Natal, Brazil; ^5^ Department of Clinical and Toxicological Analyses, Federal University of Rio Grande do Norte, Natal, Brazil; ^6^ Department of Microbiology and Parasitology, Federal University of Rio Grande do Norte, Natal, Brazil

**Keywords:** NLRP3 inflammasome, Toll-like receptor, Nod-like receptor, mice, *Trypanosoma cruzi*, innate immune receptors, virulence

## Abstract

Resistance or susceptibility to *T. cruzi* infection is dependent on the host immunological profile. Innate immune receptors, such as Toll-like receptors (TLRs/TLR2, TLR4, TLR7, and TLR9) and Nod-like receptors (NLRs/NOD1 and NLRP3 inflammasome) are involved with the resistance against acute experimental *T. cruzi* infection. Here, we evaluated the impact of *T. cruzi* virulence on the expression of innate immune receptors and its products in mice. For that, we used six *T. cruzi* strains/isolates that showed low (AM64/TcIV and 3253/Tc-V), medium (PL1.10.14/TcIII and CL/TcVI), or high (Colombian/Tc-I and Y/TcII) virulence and pathogenicity to the vertebrate host and belonging to the six discrete typing units (DTUs)—TcI to TcVI. Parasitemia, mortality, and myocarditis were evaluated and correlated to the expression of TLRs, NLRs, adapter molecules, cytokines, and iNOS in myocardium by real time PCR. Cytokines (IL-1β, IL-12, TNF-α, and IFN-*γ*) were quantified in sera 15 days after infection. Our data indicate that high virulent strains of *T. cruzi*, which generate high parasitemia, severe myocarditis, and 100% mortality in infected mice, inhibit the expression of TLR2, TLR4, TLR9, TRIF, and Myd88 transcripts, leading to a low IL-12 production, when compared to medium and low virulent *T. cruzi* strains. On the other hand, the high virulent *T. cruzi* strains induce the upregulation of NLRP3, caspase-1, IL-1β, TNF-α, and iNOS mRNA in heart muscle, compared to low and medium virulent strains, which may contribute to myocarditis and death. Moreover, high virulent strains induce higher levels of IL-1β and TNF-α in sera compared to less virulent parasites. Altogether the data indicate that differential TLR and NLR expression in heart muscle is correlated with virulence and pathogenicity of *T cruzi* strains. A better knowledge of the immunological mechanisms involved in resistance to *T. cruzi* infection is important to understand the natural history of Chagas disease, can lead to identification of immunological markers and/or to serve as a basis for alternative therapies.

## Introduction

The experimental infection of mice with different strains of *Trypanosoma cruzi* (*T. cruzi*) can range from asymptomatic to lethal infections. The susceptibility or resistance is determined by characteristics inherent to the parasite and host. Tropism, virulence, inoculation, and genetics are important characteristics of the parasite (Dias et al., 2000; Guedes et al., 2007; Gutierrez et al., 2009). *T. cruzi* strains are grouped in seven discrete type units (DTU) TcI–TcVI and TcBat, which present great variability in virulence and pathogenicity in the vertebrate host (Zingales et al., 2009; Zingales et al., 2012; Zingales, 2018).

Initial activation of immune response against *T. cruzi* occurs through the recognition of molecular patterns present in parasites by pattern recognition receptors (PRRs) (Bartholomeu et al., 2008; Oliveira et al., 2010; Rodrigues et al., 2012). Toll-like receptors (TLRs) and Nod-like receptors (NLRs) are important PRRs and play a fundamental role in modulating the immune response against *T. cruzi* (Dutra et al., 1994; Teixeira et al., 2002; Gomes et al., 2003). Several *T. cruzi* molecules have been identified as TLR agonists, such as, glycosylphosphatidinositol (GPI) which anchors activating TLR2 (Campos et al., 2001); glycoinositolphospholipid (GIPL) which induces the NF-*κ*B (nuclear factor-*κ*B) *via* TLR4 activation (Oliveira et al., 2004); RNA and DNA which can induce the activation of TLR7 and TLR9 (Koga et al., 2006; Caetano et al., 2011). The molecules present in *T. cruzi* that would be NLR agonists have not been identified yet. Parasite is recognized by different receptors and will be phagocytosed by macrophages, leading to the production of inflammatory cytokines such as interleukin-12 (IL-12) (Silva et al., 1995; Aliberti et al., 1996), which acts on natural killer (NK) cells, activating the production of more IL-12 and IFN-*γ*. The IFN-*γ* (Silva et al., 1992; Abrahamsohn and Coffman, 1996) produced, together with tumor necrosis factor alpha (TNF-α) (Silva et al., 1995; Bahia-Oliveira et al., 1998), activates the enzyme inducible nitric oxide synthase (iNOS), leading to nitric oxide (NO) synthesis, which acts to limit the intracellular multiplication of the parasite (Reis et al., 1997). On the other hand, cytokines, such as IL-10 and TGF-*β* inhibit iNOS, restricting the inflammation but allowing parasite replication (Silva et al., 1991; Holscher et al., 1998).

*Trypanosoma cruzi* experimental infection in TLR-deficient mice showed that TLR2 (Bafica et al., 2006), TLR4 (Oliveira et al., 2010), TLR7 (Caetano et al., 2011), and TLR9 (Bartholomeu et al., 2008) play a role in host resistance (Oliveira et al., 2010; Rodrigues et al., 2012). Some receptors, such as TLR2 and TLR9, act synergistically in helping to control the infection (Bafica et al., 2006). TLR3 does not appear to be involved in resistance in experimental models (Caetano et al., 2011), but TRIF^−/−^ and MyD88^−/−^ mice exhibit increased parasitism and mortality to *T. cruzi* infection (Campos and Gazzinelli, 2004; Koga et al., 2006; Bafica et al., 2006; Oliveira et al., 2010). Bone marrow-derived macrophages from NOD1 knockout mice exhibited reductions in NF-*κ*B and its products, failing to control the parasite even in the presence of IFN-*γ* (Silva et al., 2010). NLRP3 inflammasome is also important in resistance to *T. cruzi* infection (Silva et al., 2013).

Although the role of TLRs and NLRs in murine *T. cruzi* infection is clear, the influence of *T. cruzi* strains with different virulence and pathogenicity profiles on the expression of these molecules and on the modulation of the host immune response leading to asymptomatic infection or mortality is still unknown. Here we showed that the increase in virulence of *T. cruzi* strains is related to TLR2, TLR4, TLR9, TRIF, and Myd88 inhibition and NLRP3, caspase-1, IL-1β, TNF-α overexpression. A better understanding of the immunological mechanisms involved in the resistance to different strains of *T. cruzi* can lead to the identification of immunological markers and serve as a basis for therapies and prophylaxis studies.

## Materials and Methods

### Biosecurity, Animals, and Ethics Statement

All experiments were conducted according to the standard biosafety and institutional security procedures established by the Internal Biosafety Commission of the Federal University of Rio Grande do Norte (CIBio-UFRN).

Male Swiss Webster mice, 6–8 weeks old, were cared for according to institutional ethical guidelines and the Ethics Committee on Animal Use (CEUA) of the Federal University of Rio Grande do Norte (UFRN). All experiments were previously approved by protocol number 017/2012 CEUA/UFRN.

Ten animals for each *T. cruzi* strain were used for parasitemia and mortality. Another 10 animals for each group were used to determine myocarditis and cardiac parasitism, to quantify innate immunity receptors and cytokines in the myocardium by PCR, and to evaluate the levels of seric cytokines. Uninfected control group was also composed of 10 mice. Experiments were carried out in duplicate.

### Parasites and Infection

Mice were infected intraperitoneally with 1 × 10^4^ trypomastigote blood forms of *T. cruzi* strains/isolates belonging to the six genetic groups (Discrete Typing Units-DTU): Colombian—TcI, isolated from blood culture from a human case in Colombia (Federici et al., 1964); Y—TcII, isolated from an acute case of human Chagas disease in city of Marilia, state of Sao Paulo, Brazil (Silva and Nussenzweig, 1953); PL 1.10.14—TcIII, isolated by xenoculture of a *Panstrongylus lutzi* in Serra Negra do Norte, state of Rio Grande do Norte, Brazil (Martins et al., 2015); AM64—TcIV, isolated from a patient in acute phase, state of Amazonas, Brazil (Teston et al., 2013); 3253—TcV, isolated from a human case in the state of Rio Grande do Norte, Brazil (Martins et al., 2015); and CL—TcVI, isolated from *Triatoma infestans* found naturally infected in the state of Rio Grande do Sul, Brazil (Brener and Chiari, 1963).

Blood trypomastigote forms of all *T. cruzi* strains used in this research were frozen in liquid nitrogen (−196°C), defrosted, inoculated, and maintained for five successive passages in Swiss mice prior to the experiments. All strains were obtained at the Laboratory of Biology of *Trypanosoma cruzi* and Chagas disease at the Federal University of Rio Grande do Norte-UFRN coordinated by professor AC. The AM-64 strain was previously obtained from Professor Max Jean de Ornelas Toledo at the Maringa State University (UEM) and the Colombian, Y, and CL strains were previously obtained from Professor Egler Chiari at the Federal University of Minas Gerais (UFMG). The PL 1.10.14 and 3253 strains were isolated by the laboratory of the Biology of *Trypanosoma cruzi* and Chagas disease—UFRN.

### Parasitemia, Survival, and Myocarditis

The parasitemia was performed using Prager (1952) and Pizzi (1953) methods modified by Brener (1962). Parasitemia was determined microscopically by examining daily the fresh blood from the tail vein of mice infected with high virulent strains (Colombian/n = 10 and Y/n = 10), medium virulent strains (PL 1.10.14/n = 10 and CL/n = 10), low virulent strains (3252/n = 10 and AM64/n = 10) from day 5 post infection during 30 days. Animal survival was monitored daily for 60 days post infection.

Myocarditis and cardiac parasitism were determined in animals infected with high virulent strains (Colombian/n = 5 and Y/n = 5), medium virulent strains (PL 1.10.14/n = 5 and CL/n = 5), low virulent strains (3252/n = 5 and AM64/n = 5) and non-infected mice (n = 10) 15 days after infection. Tissue fragments of the heart were fixed in a 10% buffered formalin solution, dehydrated, cleared, and embedded in paraffin. Mononuclear inflammatory cells and amastigote nests were counted in thirty microscopic fields of 53,333.4 μm^2^/image, 4 μm-thick sections and stained with hematoxylin–eosin (HE) from each mouse, giving a total of 1.6 × 10^6^ μm^2^ of myocardium analyzed area. Images were obtained in an optical microscope (Olympus BX51), at a final magnification of ×400 and analyzed using the ImageJ program.

### Real Time PCR

Fragments of the heart tissue from mouse infected with each *T. cruzi* strain (n = 10), 15 days after inoculation, were collected, and total RNA was extracted using TRIzol reagent (Invitrogen™, Carlsbad, CA, USA) and SV Total RNA Isolation System kit (Promega, Madison, WT, USA). Purified RNA was stored at −80°C. RNA concentration and quality were analyzed using Nanodrop 2000 (Thermo Scientific, Waltham, MA, USA). cDNA was synthesized from 2 μg of total RNA using the High Capacity cDNA Reverse Transcription kit (Applied Biosystems, USA). The mRNA expression levels were detected by real-time PCR (qPCR) using Fast SYBR Green^®^ Master Mix (Applied Biosystems, USA) according to the instructions of the manufacturer and specific primers (**Table 1**), which were obtained by the Primer Express software (Applied Biosystems, USA). Cycles of amplification were performed in a 7500 Fast Real-Time PCR System (Applied Biosystems) using 96 well plates (MicroAmp^®^, Applied Biosystems, USA) and the standard PCR conditions consisted in an initial denaturation for 2 min at 50°C and at 95°C for 10 min followed by 40 cycles at 94°C for 30 s, variable annealing primer temperature (**Table 1**) for 30 s, and 72°C for 1 min. The determination of mRNA expression levels of innate immune receptors (TLR1, TLR2, TLR3, TLR4, TLR5, TLR6, TLR7, TLR8, TLR9, NOD1, NOD2, and NLRP3), signaling molecules (Myd88, TRIF, RIP2, ASC, and Caspase-1), cytokines (IL-1β, IL-6, IL-10, IL-12p35, IL-12p40, IL-18, IFN-*γ*, and TNF-α) and iNOS was carried out from the normalization of the result compared to the expression of the constitutive gene glyceraldehyde 3-phosphate dehydrogenase (GAPDH) using the 2^–ΔΔCt^ formula.

**Table 1:** Sequence of the used specific starters in the reactions of PCR in real time.

Iniciadores	Sense	Antisense
GAPDH	TGCAGTGGCAAAGTGGAGAT	CGTGAGTGGAGTCATACTGGAA
TLR1	TCTCTTCGGCACGTTAGC	CGTAAGAAATAAGAGCAGCCC
TLR2	CGAGTGGTGCAAGTACG	GGTAGGTCTTGGTGTTCATTATC
TLR3	GGTGGTCCCGTTAATTTCCT	CCCGAAAACATCCTTCTCAA
TLR4	CCTCTGCCTTCACTACAGAGACTTT	TGTGGAAGCCTTCCTGGATG
TLR5	CGCACGGCTTTATCTTCTCC	GGCAAGGTTCAGCATCTTCAA
TLR6	CCGGTGGAGTACCTCAAT	TCAGCAAACACCGAGTATAGC
TLR7	TGGAAATTTTGGACCTCAGC	TTGCAAAGAAAGCGATTGTG
TLR8	CACGTGTGACATAAGTGATTTTCG	TTTGATCCCCAGGATTGGAA
TLR9	CTGCCGCTGACTAATCTG	CTGAAATTGTGGCCTATACCC
NOD1	GGACAACTTGCTGGAGAAT	CTGCAGCACGTAGAGGAA
NOD2	CTTCATTTGGCTCATCCGTAG	CTGGAGATGTTGCAGTACAAAG
NALP3	AGCCTTCCAGGATCCTCTTC	GGGCAGCAGTTTCTTTC
TRIF	ATTTCAGGTGCCCGGGCGTG	TTTGCCGCTCTGCCTCCAGC
MyD88	TGATGCGGAGCCAGATT	GAGGAGGCATGTGTGTACT
RIP2	GGAGGAACAATCATCTATATGCC	ATGATCTGCAAAGGATTGGT
ASC	AAGCTGCTGACAGTGCAAC	GCCACAGCTCCAGACTCTTC
Caspase-1	AGATGGCACATTTCCAGGAC	CCTCCAGCAGCAACTTC
IL-1β	GCAACTGTTCCTGAACTCAACT	ATCTTTTGGGGTCCGTCAACT
IL-6	CCATCCAGTTGCCTTCTTG	AAGTGCATCATCGTTGTTCATAC
IL-10	TGGACAACATACTGCTAACC	GGATCATTTCCGATAAGGCT
IL-12p35	TCTCTGGACCTGCCAGGTGT	CCTGTTGATGGTCACGACGCG
IL-12p40	CAACATCAAGAGCAGTAGCAG	TACTCCCAGCTGACCTCCAC
IL-18	GTGAAGTAAGAGGACTGGCTGTG	TTTTGGCAAGCAAGAAAGTGT
TNF-α	TGTGCTCAGAGCTTTCAACAA	CTTGATGGTGGTGCATGAGA
IFN-γ	GCATCTTGGCTTTGCAGCT	CCTTTTTCGCCTTGCTGTTG
iNOS	CGAAACGCTTCACTTCCAA	TGAGCCTATATTGCTGTGGCT

### Cytokine Quantification

Cytokine production was assayed in mice sera infected with high virulent strains (Colombian/n = 5 and Y/n = 5), medium virulent strains (PL 1.10.14/n = 5 and CL/n = 5), low virulent strains (3252/n = 5 and AM64/n = 5) 15 days after infection and a non-infected control group (n = 10). The ELISA sets were IL-1β, IL-12 (p70), IFN-*γ*, and TNF-α (BD OpTEIA, BD Bioscience, San Diego, CA), and procedures were performed according to the instructions of the manufacturer. Briefly, microwells were coated with 100 μl of Capture Antibody (anti-IL-1β, anti-IL-12, anti-IFN-*γ*, and anti-TNF-α) and incubated overnight at 4°C. Plates were washed three times and blocked with assay diluent at room temperature for 1 h. Microplates were washed three times and 100 μl of each standard, sample, and control were pipetted into appropriate wells, incubated for 2 h at room temperature. Three washes were performed, and 100 μl detection antibody (anti-IL-1β/biotin, anti-IL-12/biotin, anti-IFN-γ/biotin and anti-TNF-α/biotin) together with avidin-HRP reagent was added to each well. Plates were incubated for 1 h at room temperature. Microplates were washed five times. Substrate solution (100 μl/TMB) was added to each well, and plate was incubated for 30 min at room temperature in the dark. Finally, added 50 μl stop solution was added to each well. Optical densities were measured at 450 ηm. Results were expressed as picograms per milliliter. The limits of sensitivity for IL-1β, IL-12, IFN-*γ*, and TNF-α assays were 10 pg/ml.

In order to express the repeatability and precision of the ELISA, the inter- and intra-assay Coefficients of Variation (CVs) were determined. Samples with known high and low concentrations of analyzed cytokines were used in all plates in duplicate. For the inter-assay CV, the following formula was used: $\frac{\left[(\bar{X}h+SDh)\times 100]+[(\bar{X}1+SD1)\times 100\right]}{2}$, where $\bar{X}h$ and $\bar{X}l$ are the average of high and low control samples, respectively; *SD*h and *SD*l are the standard deviation of high and low control samples, respectively. Inter-assay CVs (n = 4 plates) were 5.7, 5.3, 6.5, and 6.1% to IL-1β, IL-12, IFN-*γ*, and TNF-α, respectively. Intra-assay CV is the average of % CV from all samples, being $\%CV=(\bar{X}+SD)\times 100$. Intra-assay CVs (n = 140 samples) were 3.9, 4.2, 4.7, and 5.1% to IL-1β, IL-12, IFN-*γ*, and TNF-α, respectively.

### Statistical Analysis

The D’Agostino–Pearson and Kolmogorov–Smirnov tests were used to verify the distribution of the data. Data are presented as the mean ± standard deviation (SD). Measurements of mRNA expression levels between all the groups were compared using Kruskal–Wallis test. Cytokines and myocarditis were compared between groups using ANOVA multiple comparisons test. Correlations were performed using the Spearman test. Differences were considered significant as follows: p < 0.05 (*), p < 0.01 (**) and p < 0,001(***). The analyses were accomplished using PRISM 9.0 software (GraphPad, CA, USA).

## Results

### Virulence of *T. cruzi* Strains in Mice

**Figure 1:**
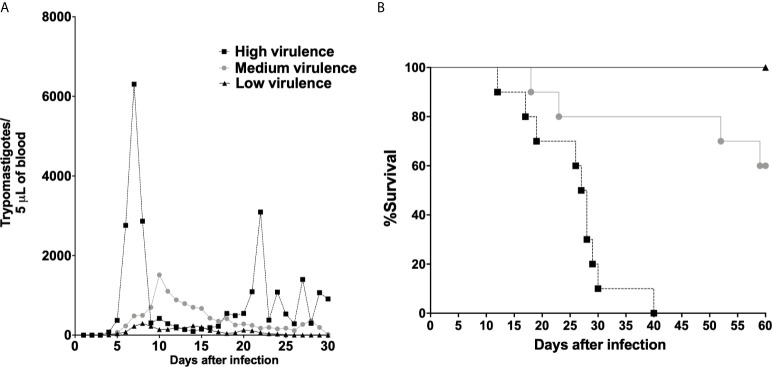
*Trypanosoma cruzi* were grouped in high, medium, and low virulent strains according to parasitemia and survival in mice. Parasitemia **(A)** and survival **(B)** in Swiss mice infected by the intraperitoneal route with 1 × 10^4^ blood trypomastigote forms of *T. cruzi* strains that showed high (Colombian + Y), medium (PL1.10.14/TcIII + CL/TcVI), and low (AM64/TcIV + 3253/TcV) virulence. The data are representative of two independent experiments (n = 10, ten animals were infected with each strain).

**Figure 2:**
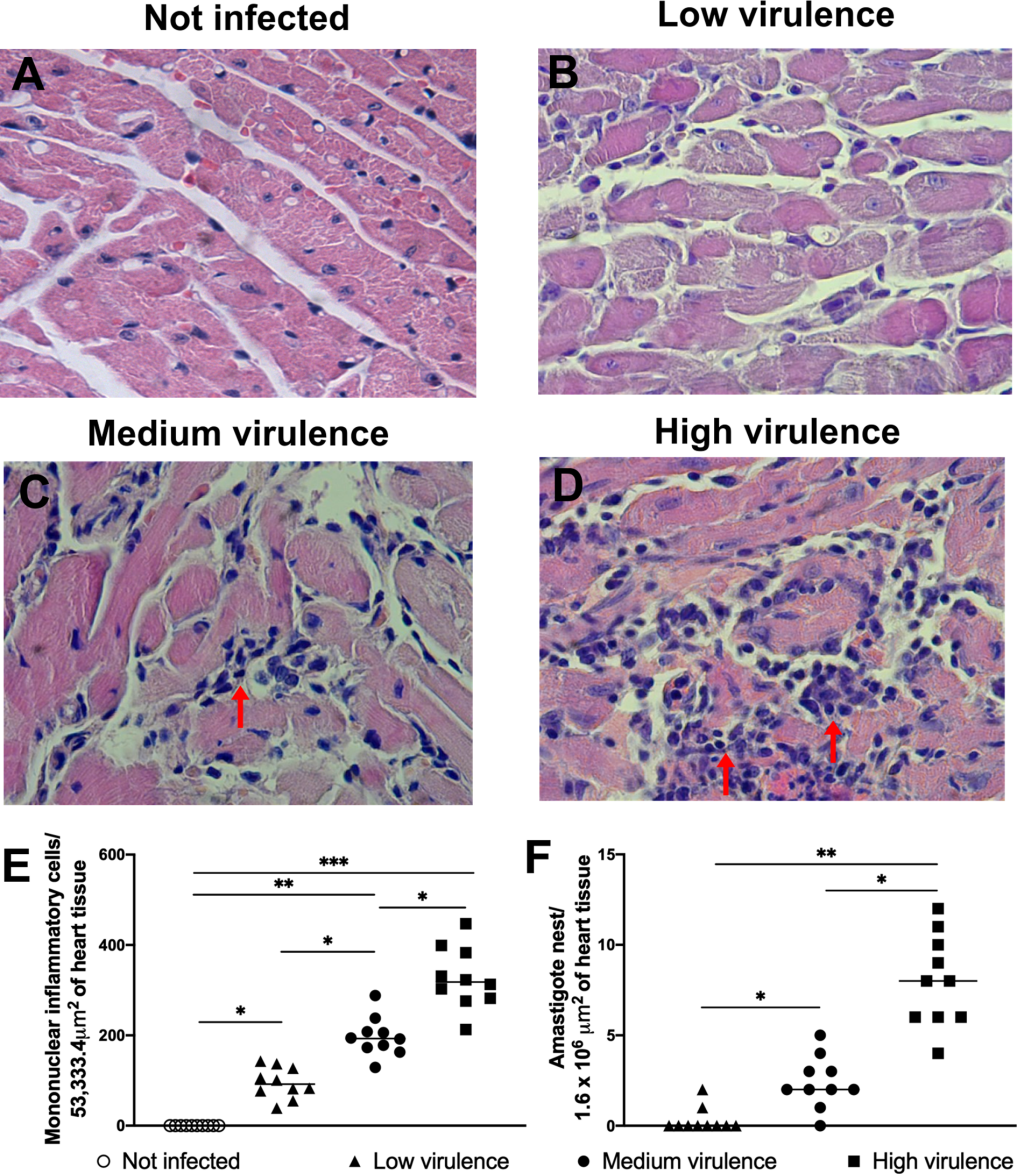
Intensity of myocarditis in the acute phase in mice is related to *Trypanosoma cruzi* strain virulence. Representative histological sections of cardiac tissue from Swiss mice, not infected **(A)** and infected by the intraperitoneal route with 1 × 10^4^ blood trypomastigote forms of *T. cruzi* strains that showed low (strains: AM64 + 3253) (n = 10) **(B)**, medium (PL1.10.14 + CL) (n = 10) **(C)** and high (Colombian + Y) (n = 10) **(D)** virulence and euthanized 15 days after infection. Quantification of inflammatory mononuclear cells **(E)** and amastigote nests **(F)** in the heart tissue of mice. Hematoxylin and eosin staining was used. ×400 magnification. Arrow indicates parasites. The results are expressed as the means ± standard errors. *p < 0.05; **p < 0.01; ***p < 0.001.

Initially, mice were infected with Colombian (Tc-I), Y (Tc-II), PL1.10.14 (Tc-III), AM64 (Tc-IV), 3253 (TC-V), and CL (Tc-VI) strains, and parasitemia and mortality were evaluated. Mice infected with Colombian and Y strains showed high levels of parasitemia and 100% mortality. Animals infected with PL1.10.14 and CL strains showed intermediate levels of parasitemia and 40% mortality. On the other hand, AM64 and 3253 generate low parasitemia and 100% of survival in infected mice (**Supplementary Figures S1A, B** **).** Thus, *T. cruzi* strains were grouped into high virulent (Colombian and Y), medium virulent (PL1.10.14 and CL), and low virulent strains. Mice infected with high virulent strains showed high levels of parasitemia and 100% mortality, while the animals infected with medium virulent strains showed intermediate levels of parasitemia and 40% mortality, and low virulent strains (AM64 and 3253) generate low parasitism load and 100% survival in mice **(** **Figures 1A, B** **).** After that we analyzed heart inflammation of mice according to its virulence and pathogenicity profile. As expected, hearts of uninfected mice showed no inflammation and hyaline degeneration **(** **Figures 2A, E** **).** Heart of mice infected with low virulent strains showed discrete myocarditis (95 ± 34 inflammatory cells/53,333.34 μm^2^) without or with low presence of amastigotes nests (0.3 ± 0.7 amastigote nests/1.6 × 10^6^ μm^2^), and few cardiac fibers had hyaline degeneration **(** **Figures 2B, E, F** **)**. The infection with medium virulent strains generated moderate myocarditis (197 ± 43 inflammatory cells/53,333.34 μm^2^), and amastigotes forms of parasite (2.4 ± 1.5 amastigote nests/1.6 × 10^6^ μm^2^) were visualized in histopathological analysis **(** **Figures 2C, E, F** **)**. Interestingly, mice infected with high virulent strains showed intense inflammatory foci (327 ± 68 inflammatory cells/53,333.34 μm^2^) in myocardium with presence of several amastigote forms of parasite (8.0 ± 2.4 amastigote nests/1.6 × 10^6^ μm^2^), and large amounts of cardiac fibers are in hyaline degeneration **(** **Figures 2D–F** **).** The results showed that myocarditis increased according to strain virulence.

### Virulence of *Trypanosoma cruzi* Strains Is Related to Low Cardiac Expression of Important TLRs Involved in the Parasitism Control

**Figure 3:**
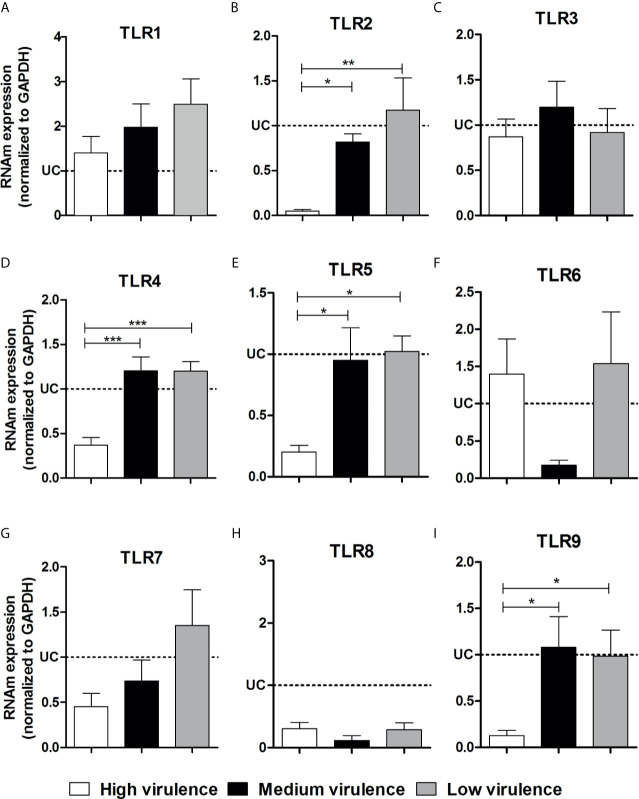
Virulence of strains is correlated with TLR2, TLR4, TLR5, and TLR9 inhibition in heart from mice. The mRNA expression levels of TLR1 **(A)**, TLR2 **(B)**, TLR3 **(C)**, TLR4 **(D)**, TLR5 **(E)**, TLR6 **(F)**, TLR7 **(G)**, TLR8 **(H)**, and TLR9 **(I)** were determined by real-time PCR in heart of Swiss mice infected by the intraperitoneal route with 1 × 10^4^ blood trypomastigote forms of *T. cruzi* strains that showed low (AM64 + 3253) (n = 10), medium (PL1.10.14 + CL) (n = 10) and high (Colombian + Y) (n = 10) virulence. The expression levels were normalized to the expression level of GAPDH. The results are expressed as the means ± standard errors. *p < 0.05; **p < 0.01; ***p < 0.001. UC, uninfected control mice (n = 10).

**Figure 4:**
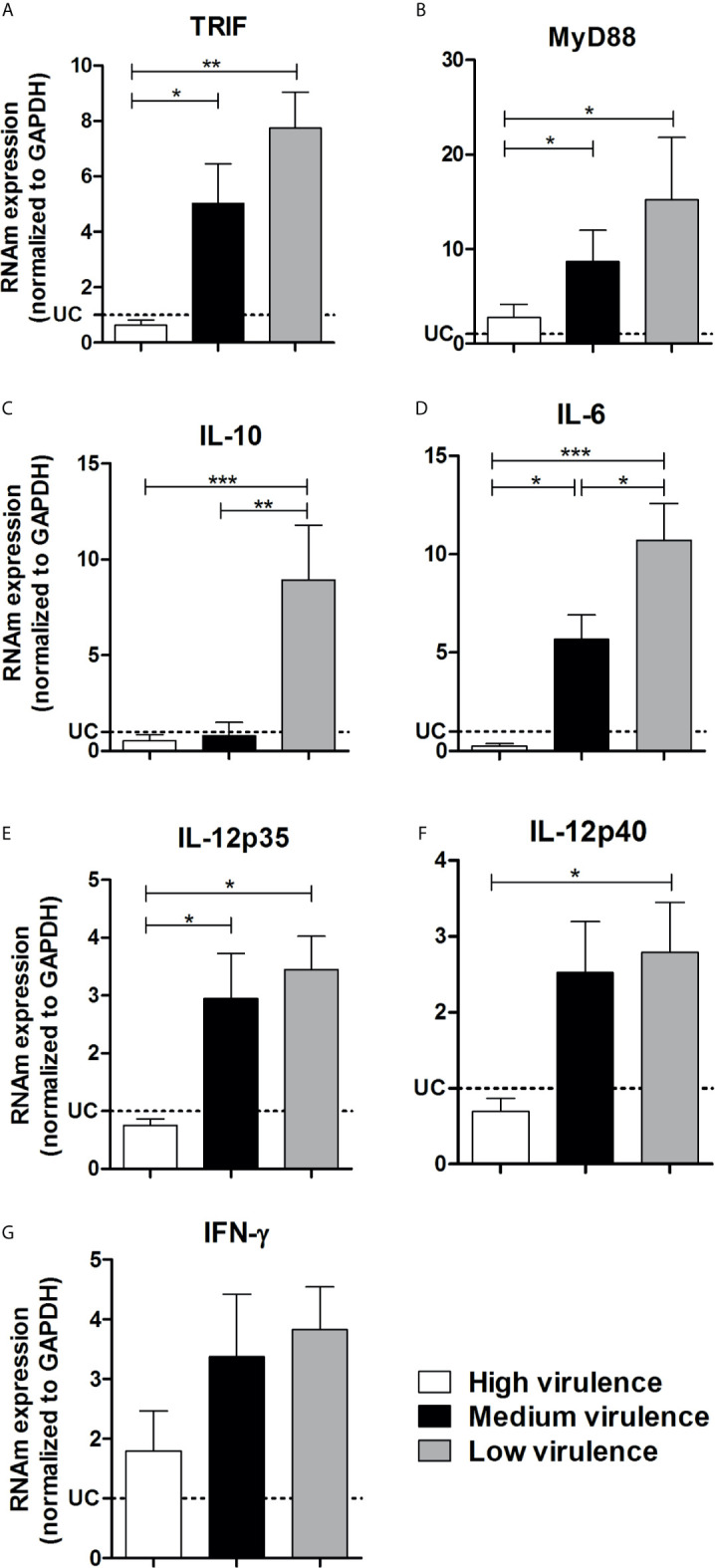
Virulence of strains is correlated with TRIF, Myd88, IL-6, IL-12 inhibition in the heart from mice.The mRNA expression levels of TRIF **(A)**, Myd88 **(B)**, IL-10 **(C)**, IL-6 **(D)**, IL-12p35 **(E)**, IL-12p40 **(F)**, and IFN-*γ* **(G)** were determined by real-time PCR in the heart from Swiss mice infected by the intraperitoneal route with 1 × 10^4^ blood trypomastigote forms of *T. cruzi* strains that showed low (AM64 + 3253) (n = 10), medium (PL1.10.14 + CL) (n = 10), and high (Colombian + Y) (n = 10) virulence. The expression levels were normalized to the expression level of GAPDH. The results are expressed as the means ± standard errors. *p < 0.05; **p < 0.01; ***p < 0.001. UC, uninfected control mice (n = 10).

In an attempt to elucidate if the differential expression of TLRs and NLRs is involved in the pathogenicity induced by different strains of the parasite, we evaluated the TLR and NLR mRNA expression in heart tissue from mice infected with high, medium, and low virulent strains of *T. cruzi*. We did not observe a difference among TLR1, TLR3, TLR6, TLR7, and TLR8 mRNA expression in heart tissue of infected mice independently of the strain evaluated **(** **Figures 3A, C, F, G, H** **)**. Interestingly, high virulent strains inhibited TLR2, TLR4, TLR5, and TLR9 mRNA expression in myocardium of infected mice, important molecules to *T. cruzi* infection resistance, when compared to medium and low virulent strains **(** **Figures 3B, D, E, I** **).** The next step was to evaluate the mRNA expression for downstream adapter molecules and cytokines in the heart tissue. High virulent strains inhibited TRIF, Myd88, IL-6, IL10, and IL-12 mRNA expression in myocardium of infected mice when compared to medium and low virulent strains **(** **Figures 4A–F** **)**. Animals infected with high virulent strains showed mRNA expression of TRIF, Myd88, IL-6, IL10, and IL-12 similar to uninfected mice. We did not observe a significant difference between IFN-*γ* transcripts in the heart from mice infected with strains that showed different profiles of virulence and pathogenicity **(** **Figure 4G** **).**

### High Virulence of *Trypanosoma cruzi* Strains Is Related to Exacerbated Expression of NLRP3, Caspase-1, IL-1β, TNF-α, and iNOS in the Myocardium

**Figure 5:**
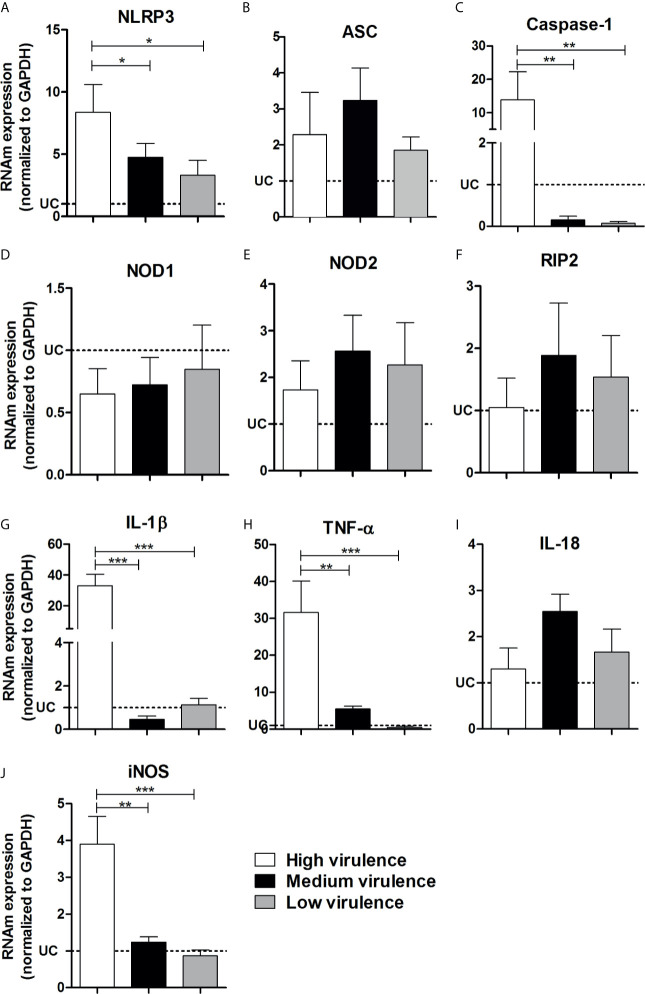
Virulence of strains is correlated with NLRP-3, caspase-1, IL-1β, TNF-α, and iNOS increase in heart from mice. The mRNA expression levels of NLRP3 **(A)**, ASC **(B)**, caspase-1 **(C)**, NOD1 **(D)**, NOD2 **(E)**, RIP2 **(F)**, IL-1β **(G)**, TNF-α **(H)**, IL-18 **(I)**, and iNOS **(J)** were determined by real-time PCR in the heart from Swiss mice infected by the intraperitoneal route with 1 × 104 blood trypomastigote forms of Trypanosoma cruzi strains that showed low (AM64 + 3253) (n = 10), medium (PL1.10.14 + CL) (n = 10), and high (Colombian + Y) (n = 10) virulence. The expression levels were normalized to the expression level of GAPDH. The results are expressed as the means ± standard errors. *p < 0.05; **p < 0.01; ***p < 0.001. UC, uninfected control mice (n = 10).

**Figure 6:**
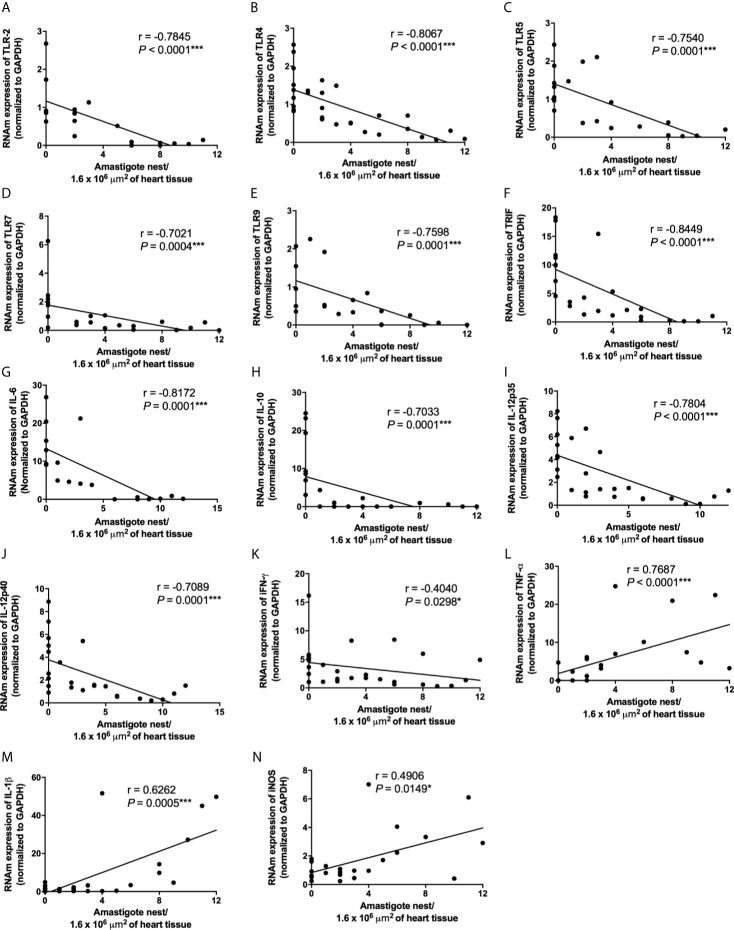
Low TLR2, TLR4, TLR5, TLR7, TLR9, TRIF, IL-6, IL-10, IL-12p35, IL-12p40, IFN-*γ* and high TNF-α, IL-1β, iNOS mRNA expression in cardiac tissue are correlated with high *Trypanosoma cruzi* virulence. The mRNA expression levels of TLR2 **(A)**, TLR4 **(B)**, TLR5 **(C)**, TLR7 **(D)**, TLR9 **(E)**, TRIF **(F)**, IL-6 **(G)**, IL-10 **(H)**, IL-12p35 **(I)**, IL-12p40 **(J)**, IFN-*γ* **(K)**, TNF-α **(L)**, IL-1β **(M)**, and iNOS **(N)** were determined by real-time PCR in cardiac tissue from Swiss mice infected by the intraperitoneal route with 1 × 10^4^ blood trypomastigote forms of *T. cruzi* strains that showed low (AM64 + 3253) (n=10), medium (PL1.10.14 + CL) (n=10), and high (Colombian + Y) (n = 10) virulence. The expression levels were normalized to the expression level of GAPDH. The results are expressed as the means ± standard errors and Spearman test was used. *p < 0.05; ***p < 0.001.

**Figure 7:**
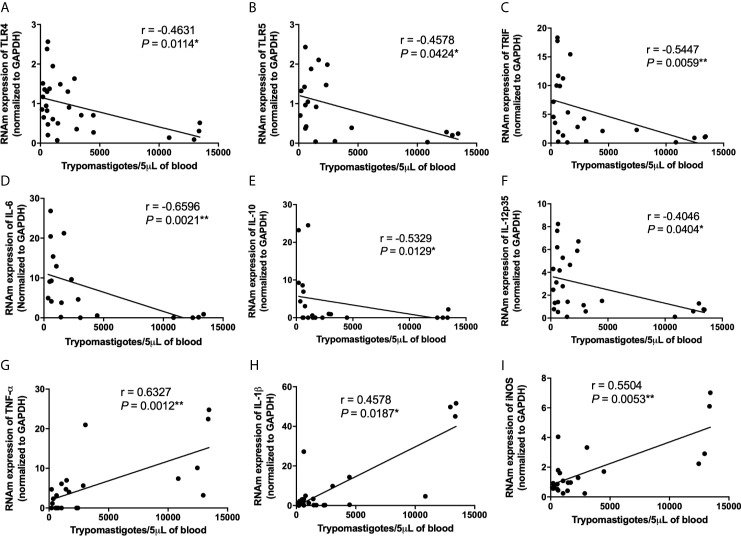
Low TLR4, TLR5, TRIF, IL-6, IL-10, IL-12p35 and high TNF-α, IL-1β, iNOS mRNA expression in cardiac tissue are correlated with high *Trypanosoma cruzi* virulence. The mRNA expression levels of TLR4 **(A)**, TLR5 **(B)**, TRIF **(C)**, IL-6 **(D)**, IL-10 **(E)**, IL-12p35 **(F)**, TNF-α **(G)**, IL-1β **(H)** and iNOS **(I)** were determined by real-time PCR in cardiac tissue from Swiss mice infected by the intraperitoneal route with 1 × 10^4^ blood trypomastigote forms of *T. cruzi* strains that showed low (AM64 + 3253) (n = 10), medium (PL1.10.14 + CL) (n = 10), and high (Colombian + Y) (n = 10) virulence. The expression levels were normalized to the expression level of GAPDH. The results are expressed as the means ± standard errors and Spearman test was used. *p < 0.05; ***p < 0.001.

Because high virulent strains induced an exacerbated inflammation in the myocardium of infected mice although they do not overexpress TLR-involved molecules or key inflammatory cytokines, we decided to analyze inflammasome-related gene expression. Interestingly, we observed an increase of NLRP3, caspase-1, IL-1β, TNF-α, and iNOS mRNA expression in the myocardium of mice infected with high virulent strain compared with animals infected with medium and low virulent strains **(** **Figures 5A, C, G, H, J** **)** We observed a similar and increased mRNA expression of ASC, NOD2, RIP2, and IL-18 in the heart of mice infected with high, medium, and low virulent strains compared to control group **(** **Figures 5B, E, F, I** **).** On the other hand, *T. cruzi-*infected mice showed a reduction of NOD1 mRNA expression in the heart when compared to uninfected animals, independent of parasite virulence **(** **Figure 5D** **).** In addition, correlation analyses showed a negative correlation between cardiac parasitism and TLR2, TLR4, TLR5, TLR7, TLR9, TRIF, IL-6, IL-10, IL-12p35, IL-12p40, and IFN-*γ* mRNA expression **(** **Figures 6A–K** and **Supplementary Table** **)**. Furthermore, we observed a positive correlation between cardiac parasitism and IL-1β, TNF-α, and iNOS mRNA expression **(** **Figures 6L–N** and **Supplementary Table** **)**. We also observed a negative correlation between TLR4, TLR5, TRIF, IL-6, IL-10, and IL-12p35 mRNA expression and parasitemia peak levels in infected mice **(** **Figures 7A–F** and **Supplementary Table** **)**. A positive correlation was observed between IL-1β, TNF-α, and iNOS mRNA expression and the number of blood trypomastigote forms **(** **Figures 7G–I** and **Supplementary Table** **)**. Altogether, these data show that during experimental *T. cruzi* infection, cardiac parasitism is inversely correlated with the expression of important TLRs to parasite control. Moreover, high virulent *T. cruzi* strains induce an overexpression of NLRP3, caspase-1, IL-1β, TNF-α, and iNOS in the myocardium.

### Increased Production of IL-1β and TNF-α During the Acute Phase Is Correlated With High Virulence of the *T. cruzi* Strains

**Figure 8:**
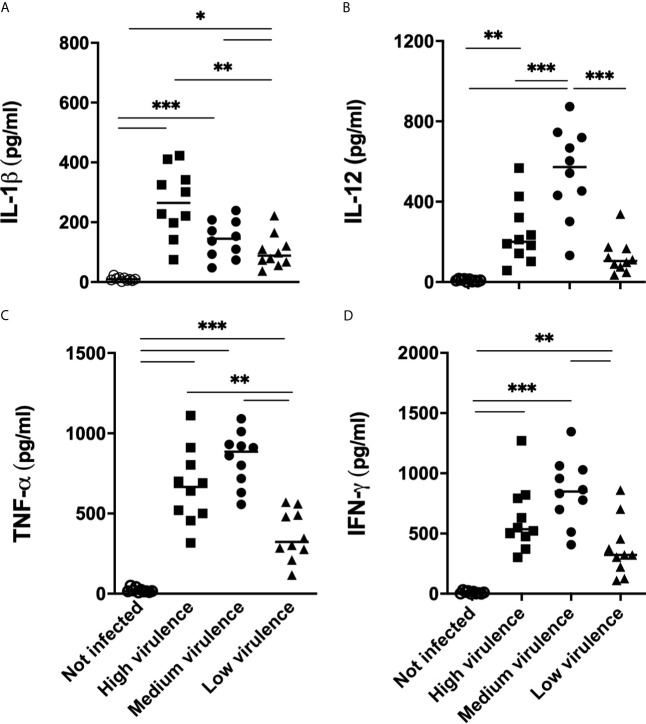
IL-1β and TNF-α in sera from mice infected with *Trypanosoma cruzi* are correlated with strain virulence (parasitemia, survival, and myocarditis). IL-1β **(A)**, TNF-α **(B)**, IL-12 **(C)** and IFN-*γ* **(D)** were quantified in the sera (15 days after infection) by ELISA from Swiss mice infected by the intraperitoneal route with 1 × 10^4^ blood trypomastigote forms of *T. cruzi* strains that showed low (AM64 + 3253) (n = 10), medium (PL1.10.14 + CL) (n = 10), and high (Colombian + Y) (n = 10) virulence. The results are expressed as the means ± standard errors. *p < 0.05; **p < 0.01; ***p < 0.001. UC, uninfected control mice (n = 10).

In an attempt to validate results of RNA expression performed by PCR, we quantified the production of cytokines in the serum of mice infected with strains of the parasite that show different degrees of virulence. *T. cruzi* infection induced the production of significant levels of IL-1β, TNF-α, IL-12, and IFN-*γ* in the serum of infected animals when compared to uninfected mice **(** **Figures 8A–D** **)**. Interestingly, greater production of IL-1β was observed in the serum of mice infected with high virulence strains when compared to mice infected with medium and low virulence strains **(** **Figure 8A** **).** Furthermore, medium virulent strains induced greater production of IL-1β than strains of low virulence **(** **Figure 8A** **).** Thus, the production of IL-1β is modulated according to the virulence of the parasite strain. High and medium virulent strains also induced high TNF-α production in sera when compared to low virulent strains **(** **Figure 8B** **).** On the other hand, mice infected with medium virulence strains showed higher levels of IL-12, IFN-*γ* in the serum, when compared to animals infected with low and high virulent strains **(** **Figures 8C, D** **)**. These data suggest that virulence of *T. cruzi* strains during the acute phase of infection in mice is related with the overexpression of inflammasome-related molecules and high levels of IL-1β and systemic TNF-α.

## Discussion

It is already well known that *T. cruzi* parasite has populations with high genetic variability and diverse biological behavior causing different clinical courses in patients and experimental infections. Clinical outcomes can range from asymptomatic to 100% lethal (Chagas, 1909; Brener and Chiari, 1963; De Araujo and Chiari, 1988; Dias, 1989; Guedes et al., 2007; Zingales et al., 2009; Guedes et al., 2009). In the present study, *T. cruzi* strains were grouped according their virulence in the vertebrate host (mouse) and evaluated according the profile of innate immune receptors (TLRs and NLRs), adapter molecules, and cytokines induced.

We initially evaluated the parasitemia and survival of *T. cruzi*-infected mice with the high (Colombian and Y), medium (CL strain and PL 1.10.14 isolate), and low virulent strains (AM64 strain and 3253 isolate). As expected, high virulent strains produced elevated parasitemia and myocarditis and generated 100% mortality in animals. Mortality of 100% in mice infected with Y and Colombian strains has already been described (De Araujo and Chiari, 1988). CL strain and PL 1.10.14 isolate induced 40% mortality; literature data demonstrated 81% mortality in C_3_H mice infected with 10^7^ metacyclic trypomastigotes of CL strain (De Araujo and Chiari, 1988). Low virulent strains (AM64 strain and 3253 isolate) showed reduced parasitemia, low heart inflammation, and 100% survival. Low levels of parasitemia and discreet myocarditis were previously described in Swiss mice infected with the same inoculum (10^4^ blood trypomastigotes) of AM-64 *T. cruzi* strain (Meza et al., 2014). However, data of parasitemia and survival in mice infected with PL 1.10.14 and 3253 are shown here for the first time.

Several studies have demonstrated the importance of TLR activation in the mechanism of protozoal infection control, such as *T. cruzi*, *T. brucei*, *Leishmania* spp., *Plasmodium* spp. and *Toxoplasma gondii* in an experimental model (Gazzinelli and Denkers, 2006). Mice are the most used experimental models. Besides having TLR11, TLR12, TLR13 that humans do not express, they have nine conserved functional members of the toll-like family (TLRs 1–9) similar to humans. Also, TLR10 is selectively expressed in humans (Kawai and Akira, 2010). However, correlation between TLR and NLR expression with virulence and pathogenicity profile generated after infection of the vertebrate host had not been evaluated yet. Interestingly, we observed that the animals infected with highly virulent strains showed inhibition in TLR2, TLR4, TLR5, and TLR9 mRNA expression in the heart. Several TLRs have been described as important molecules in the resistance to *T. cruzi* experimental infection, such as TLR2 (Bafica et al., 2006), TLR4 (Oliveira et al., 2010), TLR7 (Caetano et al., 2011), and TLR9 (Bartholomeu et al., 2008). Mice deficient of these receptors are more susceptible to infection presenting higher parasitism and mortality. These molecules are activated by parasite PAMPs such as GPI anchors, GIPL, and nucleic acids (Campos et al., 2001; Koga et al., 2006; Oliveira et al., 2010; Caetano et al., 2011), stimulating downstream adapter molecules and inducing proinflammatory cytokine production. In addition, analysis of mRNA expression of adapter molecules involved in TLR signaling pathways demonstrated that infection with highly virulent *T. cruzi* strains induced inhibition in the expression of TRIF and MyD88 in the myocardium of infected animals. Animals deficient of TRIF and MyD88 infected with *T. cruzi* are more susceptible to infection, with higher parasitism and low macrophage activation, resulting in a low production of nitric oxide (Campos and Gazzinelli, 2004; Koga et al., 2006; Bafica et al., 2006; Oliveira et al., 2010). On the other hand, medium and low virulent strains showed greater expression of these innate immunity receptors involved in the host resistance to *T. cruzi* infection when compared to high virulent strains.

In this study, inflammasome pathway analysis showed that infection with virulent strains of *T. cruzi* leads to high increase in the expression of NLRP3, caspase-1 and IL-1β in the myocardium. The high expression of these molecules may be related to the induction of inflammatory cytokines and the consequent production of nitric oxide, causing an intense inflammatory process, tissue damages, and early mortality in mice. In contrast, animals infected with strains that show low virulence showed slight enhancement of NLRP3, caspase-1, IL-1β and discreet myocarditis. Previous studies have shown that NLRP3 and caspase-1 deficient mice, experimentally infected with *T. cruzi* Y strain, have deficit NO production, which is crucial for parasite clearance, while the excess production of these molecules can generate deleterious effects on the host (Goncalves et al., 2013; Silva et al., 2013).

In this study, animals infected with highly virulent and pathogenic strains (Colombian and Y) presented a high mRNA expression of IL-1β and TNF-α in the myocardium and high serum concentrations of these cytokines. This exacerbated profile of proinflammatory cytokines is associated with high production of NO, which helps in parasitism control and can cause intense tissue destruction contributing to animal mortality. High TNF-α production may exhibit protective roles, activating macrophages but causing tissue damage generating deleterious effects on the host (Truyens et al., 1999; Holscher et al., 2000; Malvezi et al., 2004). The high expression of IL-1β is observed in patients with cardiac form of Chagas disease, and its action is correlated to cardiac hypertrophy and inhibition of fibroblast proliferation, indicating the IL-1β role in cardiac remodeling (Patten et al., 1996; Lachtermacher et al., 2010; Sousa et al., 2014). On the other hand, IL-1β showed antiparasitary action leading to TNF-α and NO production in the culture of murine cardiomyocytes infected by *T. cruzi* (Postan et al., 1999). The high production of NO by the enzyme iNOS has been associated with death of murine cardiomyocytes by triggering a programmed cell death process (Pinsky et al., 1999). Furthermore, culture of murine macrophages and cardiomyocytes with combinations of IL-1β, TNF-α, and IFN-*γ* results in death of these cells, and the lethal effects are blocked by using iNOS inhibitors or adding TGF-β (Pinsky et al., 1999).

Our data also showed that the infection with high virulent and pathogenic strains led to reduced expression of IL-6 and IL-12. These cytokines play an important role in the control of parasite replication; IL-6 deficient animals show higher parasitemia and early mortality (Muller et al., 2001; Gao and Pereira, 2002). These results suggest that deficient expression of TLR2, TLR4, TLR9 may impact on the reduced production of IL-6 and IL-12, exacerbating parasite replication and host death. In addition, a negative correlation was observed between mRNA expression of TLR4, TLR5, IL-6, and parasitemia levels. On the other hand, strains with low virulence and pathogenicity induced high expression of important TLRs and cytokines in the control of *T. cruzi* infection. In addition to regulation (inhibition or stimulation) of TLRs and NLRs, qualitative and quantitative differences in cardiac inflammatory infiltrate differentially induced by strains with high, medium, and low virulence also influence the gene expression of molecules involved in the innate immune response in cardiac tissue.

Altogether, our findings suggest that virulence of *T. cruzi* strains is related to the inhibition of innate immune receptors, adapter molecules, and cytokines important to parasitism control (TLR4, TLR9, TRIF, Myd88, IL-6, and IL-12) associated with increased expression of molecules that contribute to myocardial inflammation, damage, and mortality (NLRP3, caspase-1, IL-1β and TNF-α). Future studies will be required to identify parasite molecules implicated in the activation of the TLR receptor signaling cascade and NLR receptor signaling pathway to understand the underlying mechanisms at the interface of the immune response of the host and virulence of *T. cruzi* and its association with the pathogenesis of Chagas disease.

## Data Availability Statement

The original contributions presented in the study are included in the article/**Supplementary Material**. Further inquiries can be directed to the corresponding author.

## Ethics Statement

The animal study was reviewed and approved by Ethics Committee on Animal Use (CEUA) of the Federal University of Rio Grande do Norte (UFRN).

## Author Contributions

TQ, MN, and PG wrote the manuscript. CA, RA-J, CB, LG, AC, MN, PG conceptualize the experiments. TQ, NP, DS, CB, RA-J, and PG performed the experiments and analyzed the data. LG, AC, MN, and PG were responsible for the funding acquisition. PG was the supervision of the research. All authors contributed to the article and approved the submitted version.

## Funding

This work was supported by the Conselho Nacional de Desenvolvimento Científico e Tecnológico (CNPq/MS/SCTIE/DECIT grant no. 466698/2014-3, MCT/CNPq) and financed in part by the Coordenação de Aperfeiçoamento de Pessoal de Nível Superior - Brasil (CAPES) - Finance Code 001. The authors (PG and MN) receives a scientific productivity scholarship from Conselho Nacional de Desenvolvimento Científico e Tecnológico (CNPq).

## Conflict of Interest

The authors declare that the research was conducted in the absence of any commercial or financial relationships that could be construed as a potential conflict of interest.

## Supplementary Material

Supplementary Figure 1 Parasitemia **(A)** and survival **(B)** in Swiss mice infected by the intraperitoneal route with 1×104 blood trypomastigotes forms of *Trypanosoma cruzi* Colombian (TcI), Y (TcII), PL1.10.14 (TcIII), AM64 (TcIV), 3253 (TcV) and CL (TcVI) strains. The data are representative of two independent experiments (n=10, ten animals were infected with each strain).
