# Supplementary figures and images for: Virulence of Trypanosoma cruzi Strains Is Related to the Differential Expression of Innate Immune Receptors in the Heart
Source: Front Cell Infect Microbiol. 2021 Jul 15;11:696719. doi: 10.3389/fcimb.2021.696719 (PMC8321543; doi:10.3389/fcimb.2021.696719)

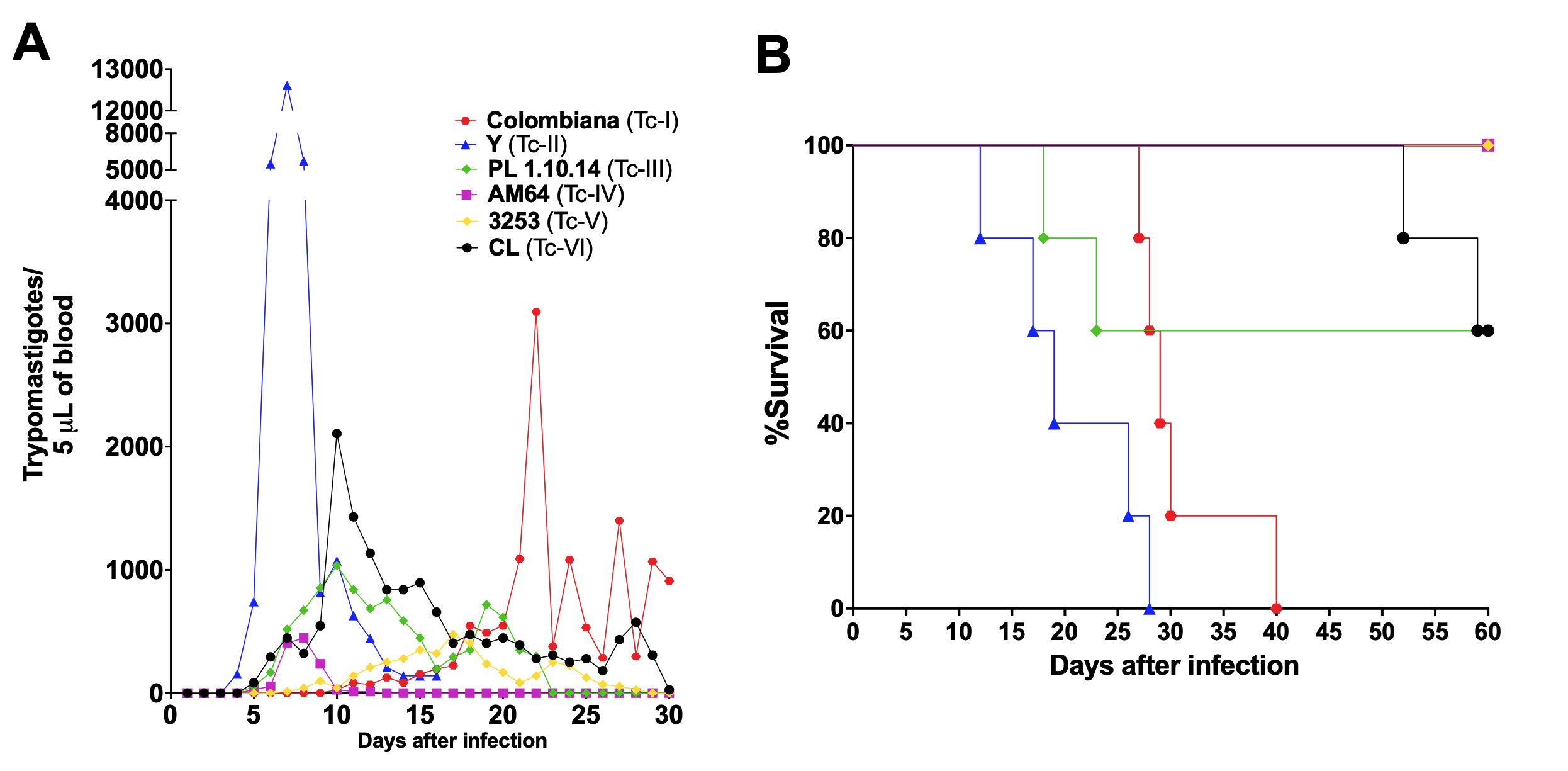

Supplement: Supplementary Figure 1 — Parasitemia (A) and survival (B) in Swiss mice infected by the intraperitoneal route with 1×10^4^ blood trypomastigotes forms of Trypanosoma cruzi Colombian (TcI), Y (TcII), PL1.10.14 (TcIII), AM64 (TcIV), 3253 (TcV) and CL (TcVI) strains. The data are representative of two independent experiments (n=10, ten animals were infected with each strain). [file Image_1.tiff]
