# Supplementary material for: Virulence of Trypanosoma cruzi Strains Is Related to the Differential Expression of Innate Immune Receptors in the Heart
Source: Front Cell Infect Microbiol. 2021 Jul 15;11:696719. doi: 10.3389/fcimb.2021.696719 (PMC8321543; doi:10.3389/fcimb.2021.696719)
Supplement: Supplementary file 2 [file Table_1.docx]

**Table.** Correlation analysis between parasitemia peak levels and heart parasitism with immunological parameters. The expression levels were normalized to the expression level of GAPDH. The results are expressed as the means ± standard errors and Spearman test was used. **p*< 0.05; ***p*< 0.01; ****p*< 0.001.

|  |  | Parasitemia peak | Heart parasitism |
| --- | --- | --- | --- |
| TLR1 | r^2^  P | 0.0082  0.9740 | -0.4611  0.0541 |
| TLR2 | r^2^  P | -0.4144  0.0902 | -0.7845  <0.0001*** |
| TLR3 | r^2^  P | 0.1180  0.6635 | -0.2083  0.4069 |
| TLR4 | r^2^  P | -0.4631  0.0114* | -0.8067  <0.0001*** |
| TLR5 | r^2^  P | -0.4578  0.0424* | -0.7540  0.0001*** |
| TLR6 | r^2^  P | -0.0791  0.7195 | 0.3206  0.1271 |
| TLR7 | r^2^  P | -0.03621  0.8866 | -0.7021  0.0004*** |
| TLR8 | r^2^  P | - 0.0507  0.8225 | 0.08014  0.7097 |
| TLR9 | r^2^  P | -0.3248  0.1623 | -0.7598  0.0001*** |
| NOD1 | r^2^  P | -0.0406  0.8290 | -0.1481  0.5117 |
| NOD2 | r^2^  P | 0.4353  0.8259 | 0.2883  0.1294 |
| NLRP3 | r^2^  P | 0.4190  0.0742 | 0.2714  0.2472 |
| TRIF | r^2^  P | -0.5447  0.0059** | - 0.8449  <0.0001*** |
| MyD88 | r^2^  P | -0.0709  0.7478 | - 0.1865  0.3720 |
| RIP2 | r^2^  P | 0.0410  0.8325 | -0.2501  0.1906 |
| ASC | r^2^  P | 0.0298  0.9034 | -0.3512  0.1185 |
| Caspase-1 | r^2^  P | 0.1657  0.4498 | 0.3577  0.0728 |
| IL-1β | r^2^  P | 0.4578  0.0187* | 0.6262  0.0005*** |
| IL-6 | r^2^  P | -0.6596  0.0021** | -0.8172  0.0001*** |
| IL-10 | r^2^  P | -0.5329  0.0129* | -0.7033  0.0001*** |
| IL-12p35 | r^2^  P | -0.4046  0.0404* | -0.7804  <0.0001*** |
| IL-12p40 | r^2^  P | -0.4015  0.0518 | - 0.7089  0.0001*** |
| IL-18 | r^2^  P | 0.02602  0.9109 | -0.2706  0.2232 |
| TNF-α | r^2^  P | 0.6327  0.0012** | 0.7687  <0.0001*** |
| IFN-γ | r^2^  P | -0.0882  0.6775 | -0.4040  0.0298* |
| iNOS | r^2^  P | 0.5504  0.0053** | 0.4906  0.0149* |
